# ReCGBM: a gradient boosting-based method for predicting human dicer cleavage sites

**DOI:** 10.1186/s12859-021-03993-0

**Published:** 2021-02-10

**Authors:** Pengyu Liu, Jiangning Song, Chun-Yu Lin, Tatsuya Akutsu

**Affiliations:** 1 Bioinformatics Center, Institute for Chemical Research, Kyoto University, Kyoto, 611-0011 Japan; 2 Monash Biomedicine Discovery Institute and Department of Biochemistry and Molecular Biology, Monash University, Melbourne, VIC 3800 Australia; 3 Institute of Bioinformatics and Systems Biology, National Chiao Tung University, Hsinchu, 300 Taiwan; 4 Center for Intelligent Drug Systems and Smart Bio-devices, National Chiao Tung University, Hsinchu, 300 Taiwan

**Keywords:** Dicer cleavage site, Gradient boosting machine, Machine learning, Cleavage sites

## Abstract

**Background:**

Human dicer is an enzyme that cleaves pre-miRNAs into miRNAs. Several models have been developed to predict human dicer cleavage sites, including PHDCleav and LBSizeCleav. Given an input sequence, these models can predict whether the sequence contains a cleavage site. However, these models only consider each sequence independently and lack interpretability. Therefore, it is necessary to develop an accurate and explainable predictor, which employs relations between different sequences, to enhance the understanding of the mechanism by which human dicer cleaves pre-miRNA.

**Results:**

In this study, we develop an accurate and explainable predictor for human dicer cleavage site – ReCGBM. We design relational features and class features as inputs to a lightGBM model. Computational experiments show that ReCGBM achieves the best performance compared to the existing methods. Further, we find that features in close proximity to the center of pre-miRNA are more important and make a significant contribution to the performance improvement of the developed method.

**Conclusions:**

The results of this study show that ReCGBM is an interpretable and accurate predictor. Besides, the analyses of feature importance show that it might be of particular interest to consider more informative features close to the center of the pre-miRNA in future predictors.

## Background

Human dicer is an RNase III enzyme that cleaves double-stranded RNA (dsRNA) and pre-miRNA into short small interfering RNA and microRNA (miRNA), respectively. It consists of six domains: Helicase, DUF283, PAZ, RNase IIIa, RNase IIIb, and dsRBD. Among these domains, the RNase IIIa domain and RNase IIIb domain cleave the 3p-arm and 5p-arm of a pre-miRNA, leading to two miRNAs.

MicroRNA (miRNA) is a class of 20-22nt long, noncoding RNA molecules. They play an important role in the posttranscriptional regulation of gene expression. Usually, one miRNA can regulate the expressions of several proteins. They are necessary for a myriad of cellular processes, such as cell differentiation, cell cycle progression, and apoptosis [1]. Several studies [2–4] show that miRNAs are related to different types of cancers such as breast, lung, and thyroid cancers. Understanding how Dicer specifically selects cleavage sites may help us interpret the effects of mutations in miRNA coding genes [5]. Therefore, it is of great interest to investigate how Dicer selects cleavage sites from the 3p-arm and the 5p-arm of a pre-miRNA.

Recently, machine learning-based approaches such as support vector machine [6–9], support vector regression [10–12], deep neural networks [13, 14] and conditional random fields [15] have been widely used for cleavage site predictions. However, these methods mainly aim at predicting protein cleavage sites. To predict human dicer cleavage sites, different feature encoding schemes and feature extraction methods are needed.

There are some existing studies on human dicer cleavage sites. Ahmed et al. [16] developed an SVM-based model (PHDCleav) of Dicer cleavage site prediction. The inputs to this model are extracted from pre-miRNA nucleotide sequences with loop/bulge structures, and the output is whether an input pattern is a correct dicer cleavage site. They demonstrated this method outperformed other approaches such as Random Forest, CART, and Naïve Bayes by computational experiments. Bao et al. [17] combined the loop/bulge size with pre-miRNA nucleotide sequences as inputs and proposed another SVM-based prediction model (LBSizeCleav). However, there are some shortcomings with these methods. First, they only considered each sequence with its loop/bulge independently. Second, their models are not explainable.

**Fig. 1:**
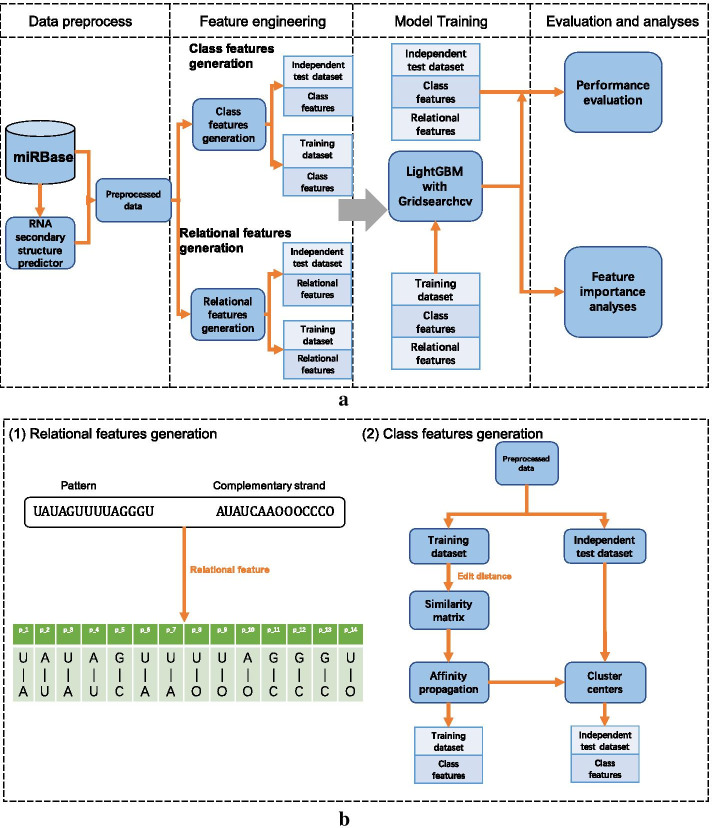
Flowchart of the data preprocessing, feature extraction, model training and evaluation of the developed ReCGBM approach. **a** Shows the design and evaluation process of ReCGBM. **b** Describes how to generate relational features and class features

To address the above issues, we propose an explainable predictor based on the gradient boosting machine [18]—ReCGBM. Our main contributions include: (i) extract relational features to combine each sequence and its complementary strand; (ii) design class features through affinity propagation, and (iii) identify some rules from the feature importance of ReCGBM. We summarize the design and evaluation process of ReCGBM in Fig. 1a.

## Methods

### Data preparation

**Fig. 2:**
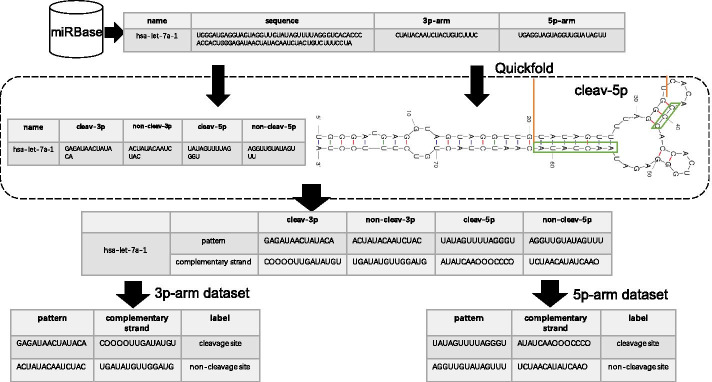
An example of data preprocessing. The sequence ’UAUAGUUUUAGGGU’ between the two red bars in the RNA structure represents the cleavage pattern of the 5p-arm. The complementary strand of this cleavage pattern is ’AUAUCAAOOOCCCO’, which can be constructed by the sequences in the green boxes and loops/bulges

In this study, we extracted the cleavage pattern and non-cleavage pattern from each pre-miRNA sequence. First, we collected 956 validated pre-miRNA sequences from miRBase (Version 22.1) [19]. To obtain the structural information for each pre-miRNA sequence, we employed quickfold [20] and RNAFold from ViennaRNA [21] to generate RNA secondary structure. We chose these two RNA secondary structure prediction methods because both are powerful tools for RNA secondary structure prediction. Besides, previous methods like PHDCleav and LBSizeCleav all employed these tools to predict RNA secondary structures. Then, we generated a cleavage pattern for each pre-miRNA sequence. Each cleavage pattern consisted of a 14 nt long sequence with the cleavage site located at the center. Finally, we extracted each non-cleavage pattern that was a 14 nt long sequence with the center 6 nt away from the corresponding cleavage site. Figure 2 illustrates how to obtain cleavage pattern and non-cleavage pattern. In this figure, the cleavage pattern of 5p-arm is the sequence between the two red bars in the structure of the pre-miRNA sequence, which is ’UAUAGUUUUAGGGU’. The non-cleavage pattern of 5p-arm is ’AGGUUGUAUAGUUU’ according to our selection rules.

To incorporate structural information of each pattern, we first obtained the structure of each pre-miRNA through quickfold or RNAFold. Then, we extracted the complementary strands for each cleavage pattern and non-cleavage pattern from the structural information of each pre-miRNA. Notice that if a nucleotide in a pattern did not have a complementary nucleotide according to the structural information, we would define it as a ’loop/bulge’. To combine ’loop/bulge’ in our data, we represented a ’loop/bulge’ as ’O’ and considered it as a complementary nucleotide of the corresponding nucleotide in a pattern. In Fig. 2, the complementary strand of the cleavage pattern of 5p-arm is given by the complementary nucleotides in the green boxes and loops/bulges (’O’). The complementary strand of the non-cleavage pattern of 5p-arm is given in the same way.

An example of data preprocessing is shown in Fig. 2. Since structural information was generated through quickfold or RNAFold, four datasets were obtained after the preprocessing: 3p-arm with quickfold structure, 5p-arm with quickfold structure, 3p-arm with RNAFold structure and 5p-arm with RNAFold structure.

### Relational features

In previous studies [16, 17], each pattern and its complementary strand were encoded by one-hot encoding separately. However, nucleotides in a pattern may also form a base pair with nucleotides in the corresponding complementary strand.

To better encode the relation between each pattern and its complementary strand, we considered relational features. For example, given a pattern ’UAUAGUUUUAGGGU’ and its complementary strand ’AUAUCAAOOOCCCO’, the relational features can be obtained according to (1) shown in Fig. 1b. An advantage of relational features is that it offers the important base-pairing information between each pattern and its complementary strand.

### Class features

Previous methods [16, 17] considered each input independently to make predictions. However, similar inputs may lead to the same prediction outputs. In this study, we made an assumption that similar inputs will lead to the same prediction results and accordingly designed the class feature, which assigned similar inputs to the same class.

**Fig. 3:**
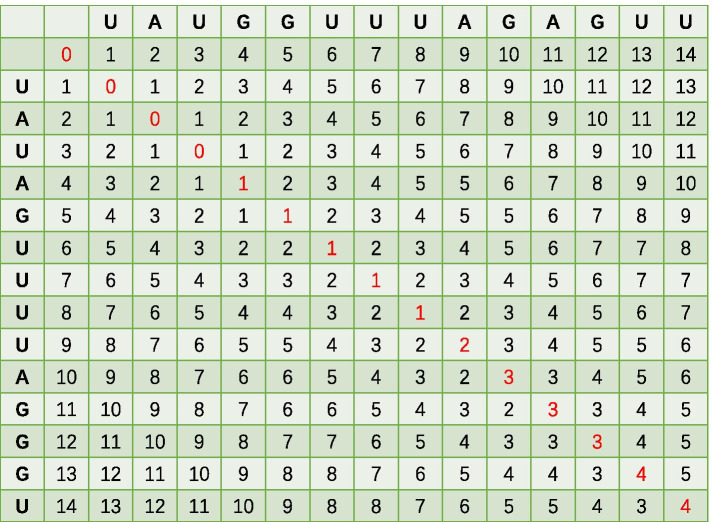
An example of calculating the edit distance by matrix

To obtain class features, we first defined the pairwise similarities between different inputs based on the edit distance (Fig. 3). Then, an unsupervised learning method — affinity propagation [22] was used to cluster the inputs to obtain the class features.

#### Edit distance

In order to measure the pairwise similarities between different sequences, we used the edit distance [23].

Given two strings *A* and *B*, suppose that the lengths of *A* and *B* are |*A*| and |*B*|, respectively. The edit distance between *A* and *B* is given by $D_{edit}(|A|, |B|)$ as follows

$$\begin{aligned}D_{edit}(i, j)= {\left\{ \begin{array}{ll} max(i,j), &{}\text {if } min(i,j)=0; \\ min{\left\{ \begin{array}{ll} D_{edit}(i-1, j) + 1 \\ D_{edit}(i, j-1) + 1 \\ D_{edit}(i-1, j-1) + 1_{a_i \ne b_j} \\ \end{array}\right. } &{} \text {otherwise.}\\ \end{array}\right. } \end{aligned}$$

where *i* represents the first *i* characters of *A* and *j* represents the first *j* characters of *B*, respectively, where $i,j \ge 1$.

$1_{a_i \ne b_j}$ is an indicator function where

$$\begin{aligned}1_{a_i \ne b_j}= {\left\{ \begin{array}{ll} 0 &{}\text {if } a_i=b_j; \\ 1 &{} \text {otherwise.}\\ \end{array}\right. } \end{aligned}$$

Since our inputs are 14 nt RNA sequences, the similarity between two sequences can be calculated by the edit distance. For example, given two sequences ’UAUAGUUUUAGGGU’ and ’UAUGGUUUAGAGUU’, the edit distance between them can be calculated through a distance matrix (Fig.  3).

As Fig. 3 shows, the edit distance between these two sequences is 4.

In this study, each input consisted of a pattern and its complementary strand. Therefore, the similarity between the two inputs *E* and *F* can be defined as follows:

$$\begin{aligned} D_{similar}(E, F) = D_{edit}(|E_1|, |F_1|) + D_{edit}(|E_2|, |F_2|) \end{aligned}$$

where $E_2$ and $F_2$ are the complementary strands of $E_1$ and $F_1$ respectively.

Given a dataset that includes *n* samples, we define a $n \times n$ similarity matrix *S* where the entry in the *i*th row and *j*th column $s(i,j) = -d_{i,j}$. Notice that $d_{i,j}$ denotes the similarity $D_{similar}(i, j)$ between the *i*th training sample and the *j*th training sample.

#### Affinity propagation

Affinity Propagation [22] is a clustering algorithm based on message passing. It identifies classes of similar inputs. Given *n* data points $x_1, \dots , x_n$, the algorithm works as follows:

- Define an $n \times n$ similarity matrix *S* with $s(i,j) = -d_{i,j}$ for $1 \le i \le n, 1 \le j \le n$. $d_{i,j}$ is the distance between $x_i$ and $x_j$;
- Define an $n \times n$ responsibility matrix *R* with $r(i,j) = 0$ for $1 \le i \le n, 1 \le j \le n$;
- Define an $n \times n$ availability matrix *A* with $a(i,j) = 0$ for $1 \le i \le n, 1 \le j\,\le\,n$;
- Iteratively execute the follow steps:
  1. Responsibility updates:
    $$\begin{aligned} r(i,k) \leftarrow s(i,k) - \max _{k^{\prime } \ne k}\{a(i, k^{\prime }) + s(i, k^{\prime })\} \end{aligned}$$
  2. Availability updates:
    $$\begin{aligned} a(i,k) &\leftarrow \min (0, r(k,k)+\sum _{i^{\prime } \in \{i,k\}} \max (0, r(i^{\prime },k))) \text { for } i \ne k \\  a(k,k) & \leftarrow \sum _{i^{\prime } \ne k}\max (0, r(i^{\prime },k)) \end{aligned}$$
  until $A+R$ remain unchanged over a number of steps, or after some predefined numbers of steps. For each point $x_i$, the data point $x_k$ that maximizes $a(i,r)+ r(i,k)$ gives us the class information of $x_i$.

We chose affinity propagation to generate class features as it only requires a few hyperparameters. More importantly, affinity propagation does not need to choose the number of classes. To employ affinity propagation, we used the edit distance to generate similarity matrices.

**Fig. 4:**
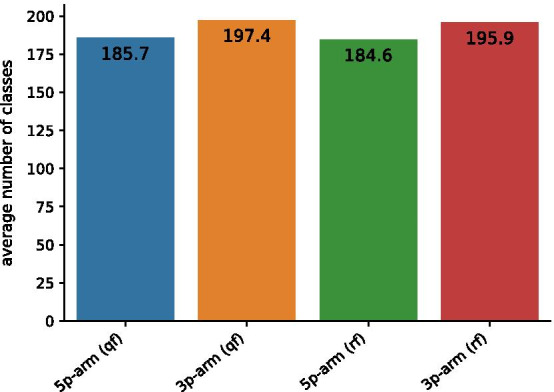
Average number of classes for different data types. The terms qf and rf represent quickfold and RNAFold, respectively

Given a training set and a test set, we first calculated the similarity matrix of the training set. Then we applied affinity propagation to the similarity matrix. The affinity propagation will assign each sample in the training set a cluster label, which is our class features. Besides, the number of clusters and the center of each cluster were also obtained from the results of affinity propagation (Fig. 4). We then measured the edit distance between each sample in the test set and these cluster centers. Finally, we assigned each test sample the same cluster label as the cluster center with the minimum edit distance. The whole procedure is given by (2) in Fig. 1b.

### LightGBM

Gradient boosting machine [18] is a machine learning algorithm that uses a group of weak prediction models (often decision trees) to make predictions. In this study, we utilized a gradient boosting machine-based framework — lightGBM.

LightGBM [24] is an efficient implementation of gradient boosting machine. It has been widely used in the field of bioinformatics and compuational biology since it has the following advantages:

- High speed and low memory cost: LightGBM uses a histogram-based algorithm [25–27]. Such algorithm can assign continuous feature values into discrete bins, thereby leading to high training speed and low memory cost.
- High accuracy: Traditional decision tree-based learning algorithms generate trees level-wise. However, lightGBM generates trees leaf-wise. This strategy usually causes lower loss than level-wise algorithms.
- Support categorical features: One-hot encoding is an efficient encoding scheme for categorical features. However, for tree-based learning algorithms, one-hot features tend to generate very unbalanced trees, which may prevent the prediction model from achieving good accuracy. Instead of one-hot encoding, lightGBM allows users to input categorical features directly to train the model, which may lead to more balanced trees and more accurate results.

We built a lightGBM-based model — termed ReCGBM with relational features and class features as inputs. The outputs were cleavage sites or non-cleavage sites.

### Evaluation metrics

To assess the performance of our prediction model, we used several different metrics including sensitivity (Sn), specificity (Sp), accuracy (Acc) and Matthews correlation coefficient (MCC):

$$\begin{aligned} Sn= & {} \dfrac{TP}{TP+FN} \\ Sp= & {} \dfrac{TN}{TN+FP} \\ Acc= & {} \dfrac{TP+TN}{TP+TN+FP+FN} \\ MCC= & {} \dfrac{TP \times TN - FP \times FN}{\sqrt{(TP+FP) \times (TP+FN) \times (TN+FP) \times (TN+FN)}} \end{aligned}$$

where *TP*, *TN*, *FP* and *FN* denote the numbers of true positives, true negatives, false positives, and false negatives, respectively.

## Results

### Predictive performance

The main goal of this paper is to develop an accurate prediction model for the Dicer cleavage sites. In this section, we show the predictive performance of ReCGBM and compare our results with other existing models.

We built prediction models for the 5p-arm dataset and 3p-arm dataset with secondary structures predicted by quickfold and RNAFold respectively.

To ensure the effectiveness of our model, we trained 10 models for each dataset, where we only considered the cases in which the affinity propagation converged. For each model, we randomly divided our preprocessed dataset into two subsets. The first subset that included 800 cleavage patterns and 800 non-cleavage patterns was used as the training set. The other subset was used as the independent test set, which included 156 cleavage patterns and 156 non-cleavage patterns. We computed the average sensitivity (Sn), specificity (Sp), accuracy (Acc), and MCC of the 10 models for each dataset.

We compared the predictive performance of ReCGBM with the existing methods, PHDCleav and LBSizeCleav. Since the performance of LBSizeCleav highly depends on the variable *k*, which represents the effect of length of loops/bulges on the kernel computation, we trained LBSizeCleav with $k=1,2,3,4,5$ as previously described [17]. All models were trained on the same 10 training sets and evaluated on the same 10 test sets for each dataset.

In order to tune the hyperparameters, we performed grid search on the training set for each models with GridSearchCV in scikit-learn [28]. For ReCGBM, we performed a grid search with $max\_depth \in [10, 20, 30, 40, 50, 60]$ , $learning\_rate \in [0.05, 0.1, 0.15]$, and $num\_leaves \in [200, 300, 400]$, respectively. For PHDCleav and LBSizeCleav-based models, we performed grid search with $C \in [1, 2, 3, 4, 5, 6, 7, 8, 9, 10]$, and $gamma \in [0.1, 0.01, 0.001]$. After the best hyperparameters were chosen, the models with the best hyperparameters were trained on each training set.

**Fig. 5:**
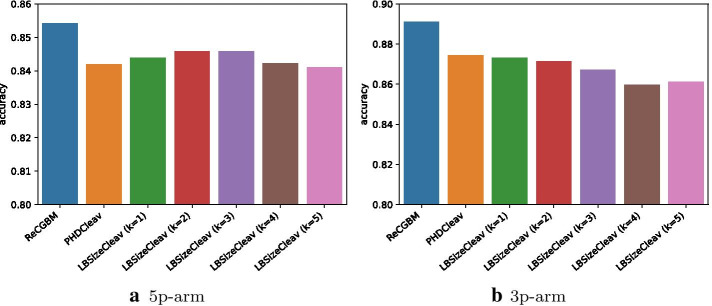
Performance comparison between different models based on the datasets with secondary structures predicted by quickfold

The predictive performance of different models is illustrated in Fig. 5 and Additional file 1: Table S1 for both the 5p-arm dataset and the 3p-arm dataset with secondary structures predicted by quickfold. For the 5p-arm dataset with secondary structures predicted by quickfold, ReCGBM achieved a sensitivity of 0.863, a specificity of 0.846, an accuracy of 0.854 and an MCC of 0.709, respectively, which outperformed all other predictors in terms of three out of the four evaluation metrics. For the 3p-arm dataset with secondary structures predicted by quickfold, ReCGBM achieved the best sensitivity, specificity, accuracy and MCC of 0.883, 0.899, 0.891 and 0.783, respectively.

**Fig. 6:**
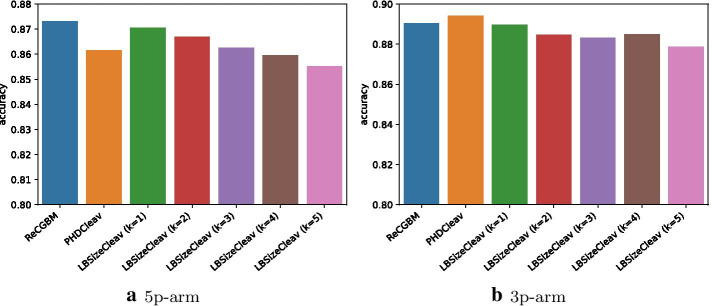
Performance comparison between different models based on the datasets with secondary structures predicted by RNAFold

Figure 6 and Additional file 1: Table S2 show the average specificity, sensitivity, accuracy, and MCC of models on both the 5p-arm dataset and the 3p-arm dataset with secondary structures predicted by RNAFold. For the 5p-arm dataset with secondary structures predicted by RNAFold, ReCGBM achieved the best specificity (0.862), accuracy (0.873) and MCC (0.747). In contrast, LBSizeCleav (k=3) achieved the best sensitivity (0.888). For the 3p-arm dataset with secondary structures predicted by RNAFold, ReCGBM achieved the best specificity (0.892), while PHDCleav achieved the best sensitivity (0.904), accuracy (0.894) and MCC (0.789).

Overall, ReCGBM outperformed the other models on three of the four datasets, highlighting the effectiveness of this model for predicting human dicer cleavage sites.

To further investigate whether different RNA secondary structures affect our prediction accuracy, we also generated relational features and class features using the RNAstructure package [29, 30]. For $\sim 70$nt RNA structure, RNAstructure predicts secondary structures of the pre-miRNA-size RNAs with the accuracy near $100\%$ .We trained 5 models for each dataset, where we only considered the cases in which the affinity propagation converged. For each model, we randomly divided our preprocessed dataset into two subsets. The first subset that included 800 cleavage patterns and 800 non-cleavage patterns was used as the training set. The other subset was used as the independent test set, which included 156 cleavage patterns and 156 non-cleavage patterns. The results are shown in Additional file 1: Table S3. The performance of ReCGBM with RNAstructure is close to the performance of ReCGBM with RNAFold.

### Affinity propagation

The goal of this experiment is to explore the relationship between class features and cleavage/non-cleavage sites. To address this, we applied affinity propagation to each training set of 3p-arm and 5p-arm with secondary structures predicted by quickfold and RNAFold. Figure 4 shows the average cluster results of 10 training sets of 3p-arm and 5p-arm with secondary structures predicted by quickfold and RNAFold.

To further investigate the relationship between the class features and the cleavage/non-cleavage sites, we defined $ratio_i(\text {cleavage})$ for each class *i* in Fig. 4 as follows:

$$\begin{aligned} ratio_i(\text {cleavage}) =\frac{N_i(\text {cleavage})}{N_i(\text {non-cleavage})+N_i(\text {cleavage})} \end{aligned}$$

where $N_i(\text {cleavage})$ and $N_i(\text {non-cleavage})$ represent the number of cleavage patterns and the number of non-cleavage patterns in class *i*, respectively. If $ratio_i(\text {cleavage})$ is close to 0, the samples in class *i* are almost non-cleavage patterns. On the other hand, if $ratio_i(\text {cleavage})$ is close to 1, the samples in class *i* are almost cleavage patterns. Classes with very high $ratio(\text {cleavage})$ and classes with very low $ratio(\text {cleavage})$ are desirable as these classes reflect that the class features have the potential to distinguish cleavage sites from non-cleavage sites.

We assume class *i* belongs to label 1, 2, 3, 4, 5 if $ratio_i(\text {cleavage}) \in [0, 0.2]$, (0.2, 0.4], (0.4, 0.6], (0.6, 0.8], (0.8, 1.0], respectively.

**Fig. 7:**
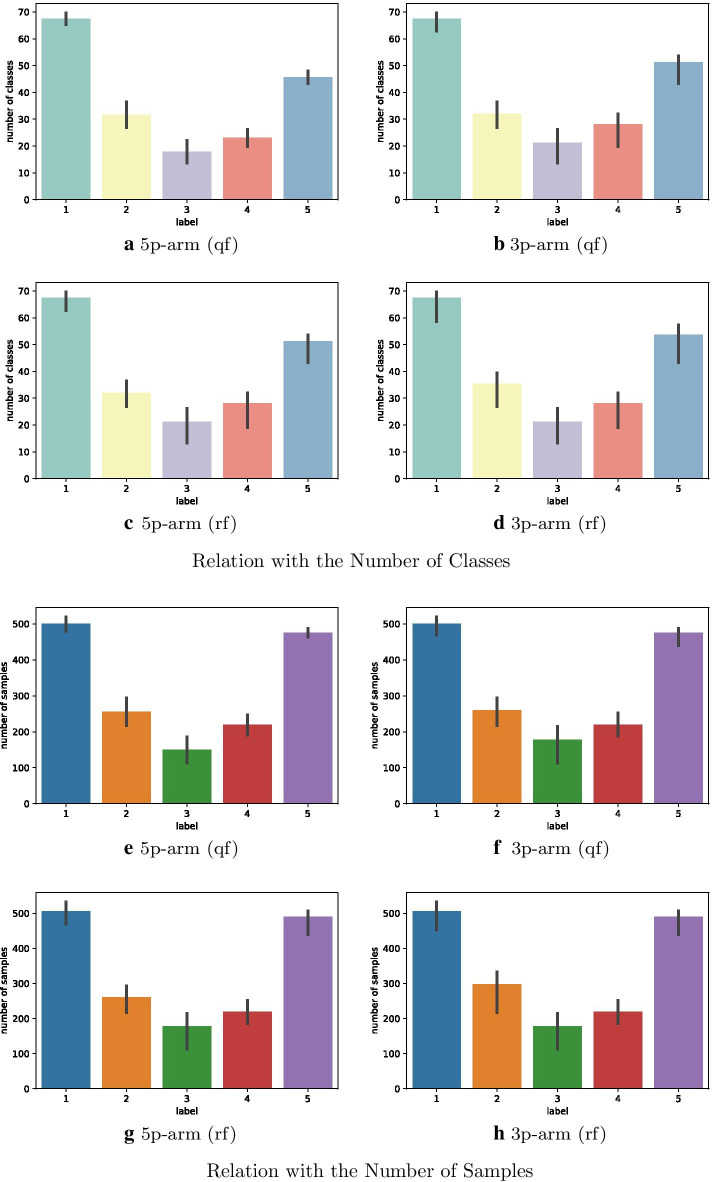
Results of affinity propagation. **a**–**d** plot the relationships between the average number of classes and different labels for the 5p-arm dataset (qf), 3p-arm dataset (qf), 5p-arm dataset (rf) and 3p-arm dataset (rf), respectively where qf represents secondary structures predicted by quickfold server and rf denotes secondary structures predicted by RNAFold. **e**–**h** show the relationships between the average number of samples and different labels for the 5p-arm dataset (qf), 3p-arm dataset (qf), 5p-arm dataset (rf) and 3p-arm dataset (rf), respectively

Figure 7a–d show the relationships between the average number of classes and each label for the 5p-arm dataset(qf), 3p-arm dataset(qf), 5p-arm dataset(rf) and 3p-arm dataset(rf) in Fig. 4, respectively. The commonality in these four figures is that the numbers of classes in label 1 and label 5 were much higher than others. Figure 7e–h describe the relationships between the average number of samples and each label for four datasets. It is obvious that the numbers of samples in label 1 and label 5 were much higher than others.

Thus, the results of affinity propagation show that the majority of classes are overwhelmed by either only cleavage sites or non-cleavage sites, which indicates that the clusters based on edit distance may improve the prediction of the cleavage site.

### Sequence logo representations

**Fig. 8:**
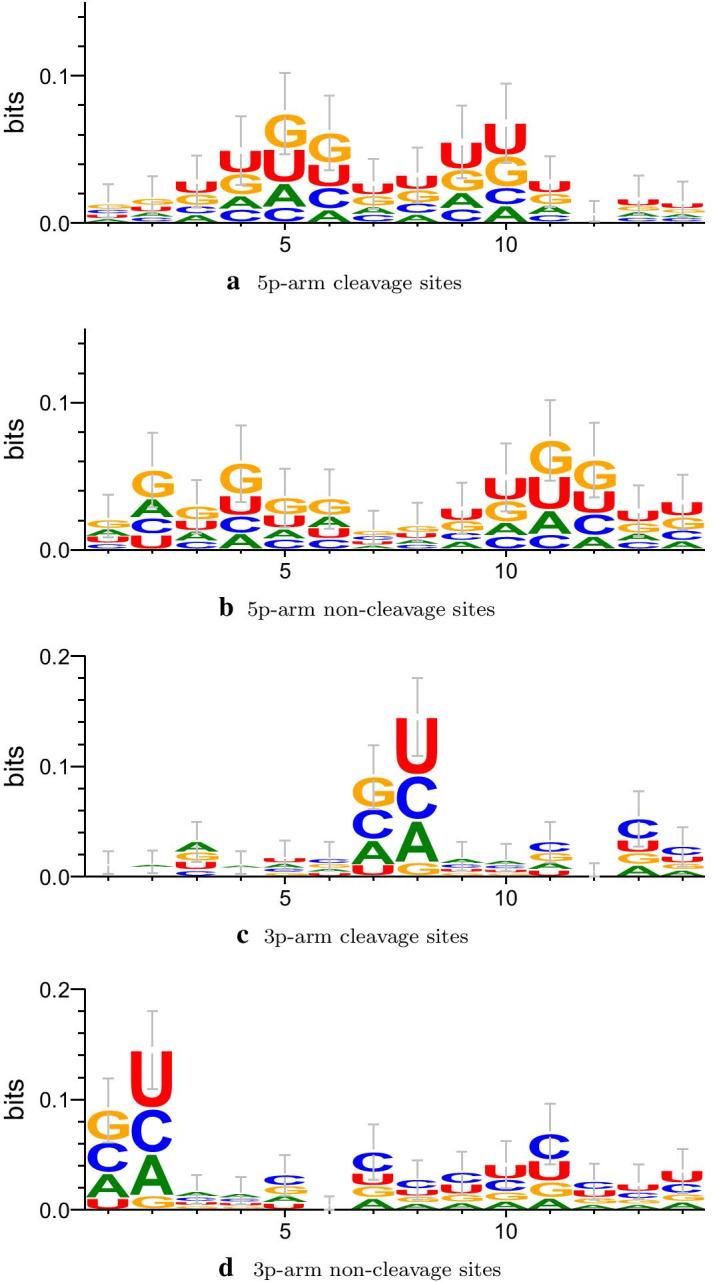
Sequence logo representations of cleavage sites and non-cleavage sites. **a** and **b** are sequence logo representations of the 5p-arm cleavage sites and the 5p-arm non-cleavage sites. **c** and **d** are sequence logo representations of the 3p-arm cleavage sites and the 3p-arm non-cleavage sites

To explore the difference between cleavage sites and non-cleavage sites at the RNA sequence level, we draw the sequence logo representations (Fig. 8) of the 5p-arm cleavage sites, 5p-arm non-cleavage sites, 3p-arm cleavage sites and 3p-arm non-cleavage sites by WebLogo 3 [31].

As can be seen from Fig. 8, the 5p-arm cleavage sites and 5p-arm non-cleavage sites show different preferences for the neighboring nucleotides. For cleavage sites, sequence motifs associated with over-represented nucleotides at the positions 3-11 (Fig. 8a) can be easily observed, while for non-cleavage sites, no specific nucleotides were found to be over-represented at the positions 7 and 8 (Fig. 8b). For the 3p-arm cleavage sites (Fig. 8c), nucleotides at two specific positions 7 and 8 showed most distinctive preferences, compared with the 3p-arm non-cleavage sites (Fig. 8d). There also exist subtle differences in other positions such as the positions 5, 9, 10, and 11 between the 3p-arm cleavage sites and non-cleavage sites. Altogether, the nucleotide preferences shown in Fig. 8 represent patterns important for distinguishing the dicer cleavage sites from non-cleavage sites.

### Feature importance

Gradient boosting machine typically uses decision trees as the base learners. An advantage of the decision tree is that it is an explainable model. Therefore, it is also possible to interpret a gradient boosting machine as an ensemble of decision trees. Fortunately, lightGBM has a built-in module that provides a score to describe the usefulness of each feature for a trained model. Here we will discuss the feature importance of ReCGBM.

Since we trained 10 models for each dataset, we list the average feature importance of the 10 trained models for each dataset.

**Fig. 9:**
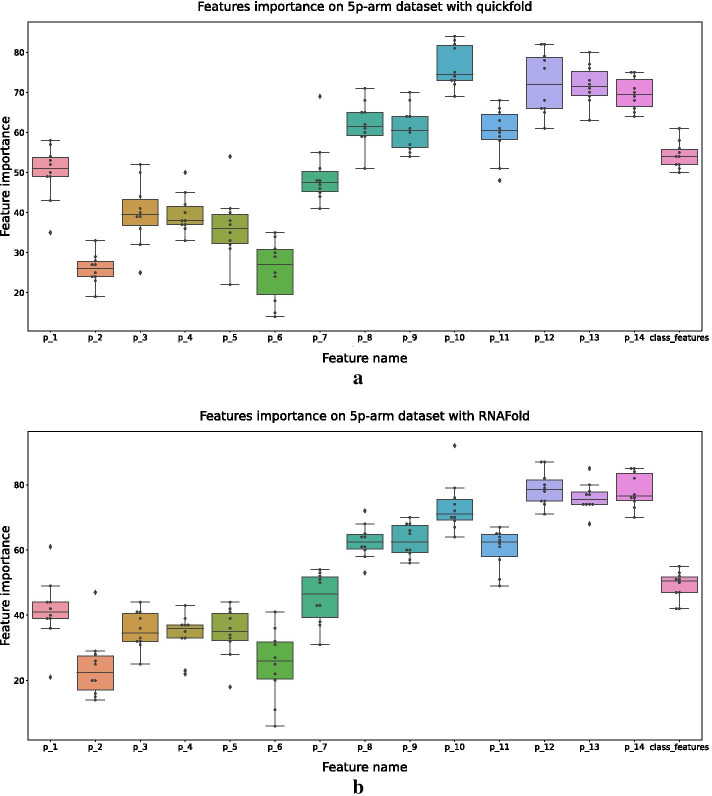
Results of feature importance on the 5p-arm datasets. The secondary structures of **a** and **b** are predicted by quickfold and RNAFold, respectively

**Fig. 10:**
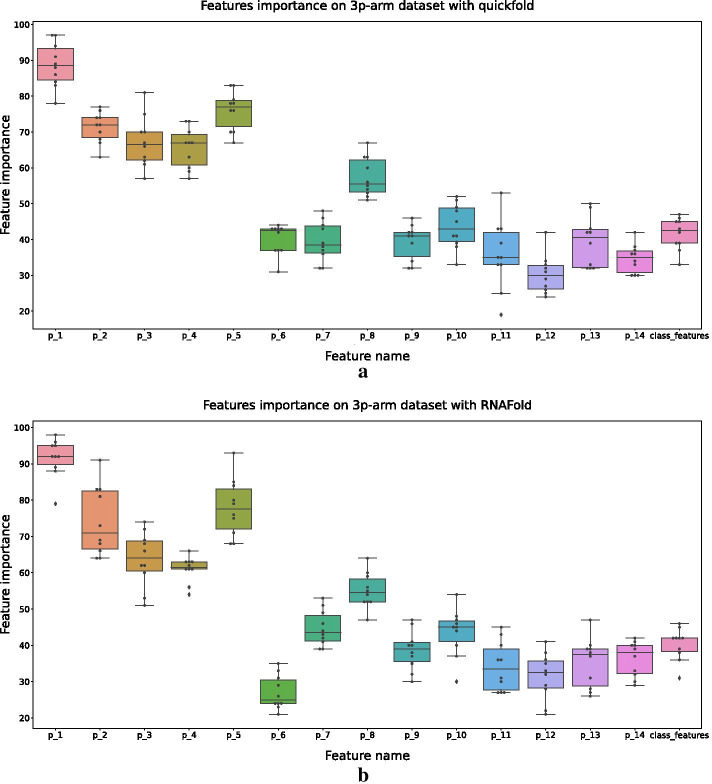
Results of feature importance on the 3p-arm datasets. The secondary structures of **a** and **b** are predicted by quickfold and RNAFold, respectively

Let $p_1$, $\dots$, $p_{14}$ denote the 14 pairs of the relational features (Fig. 1b1). Figures 9 and 10 show the results of feature importance for different datasets.

Figure 9 gives the feature importance of the 5p-arm datasets with the secondary structures predicted by quickfold and RNAFold, respectively. It can be seen that $p_8$, $\dots$, $p_{14}$ were more important than $p_1$, $\dots$, $p_7$. Additional file 1: Figure S1a and S1b shows the relationships between $p_8$, $\dots$, $p_{14}$ and $p_1$, $\dots$, $p_7$ on 5p-arm dataset directly.

Figure 10 shows the feature importance of the 3p-arm datasets with the secondary structures predicted by quickfold and RNAFold, respectively. For the 3p-arm datasets, $p_1$, $\dots$, $p_7$ were more important than $p_8$, $\dots$, $p_{14}$. Additional file 1: Figure S1c and S1d shows the relationships between $p_8$, $\dots$, $p_{14}$ and $p_1$, $\dots$, $p_7$ on the 3p-arm dataset directly.

## Discussion

Although ReCGBM showed a better performance on three of the four datasets, PHDCleav achieved the best performance on the 3p-arm dataset with the secondary structures predicted by RNAFold. There are several potential reasons. First, the performance of ReCGBM highly depends on the predicted secondary structures. To obtain relational features and class features, the secondary structure information is necessary. However, the secondary structure generated by quickfold or RNAFold may include several structures. In ReCGBM, only one structure can be included in our input features, which might affect the prediction performance.

Second, some class features generated by the affinity propagation may not be accurate. As shown in Fig. 4, the number of classes given by the affinity propagation for each dataset was over 180. However, the number of samples in each dataset was 1912. Thus such classes may exist: the distance between each sample in this class may not be so close. The distance between samples in this class and samples in other classes is relatively farther. The affinity propagation may fail to find data that are ’close enough’ to each sample in such classes. By ’close enough’ we mean different samples are close enough such that they share some common properties.

The feature importance of ReCGBM also shows some connections between the RNA secondary structures and human dicer cleavage site prediction. Considering the position of relational features in the secondary structure of pre-miRNA (Additional file 1: Figure S2), $p_8$, $\dots$, $p_{14}$ of 5p-arm and $p_1$, $\dots$, $p_7$ of 3p-arm are closer to the center of the pre-miRNA. Therefore, relational features close to the center of pre-miRNA may contribute more to human dicer cleavage sites prediction.

Another observation is that class features are more important in the 5p-arm datasets, which can be concluded based on the results shown in Figs. 9 and 10.

## Conclusions

In summary, we have introduced a lightGBM-based model—termed as ReCGBM for accurate prediction of human dicer cleavage sites and analyzed the feature importance of ReCGBM. Computational experiments demonstrated the effectiveness of this model. However, possible improvements can be achieved in the future. First, affinity propagation finds the number of clusters automatically. However, in some cases it is hard to converge on small datasets. Thus, an easy-to-converge cluster algorithm is desirable. Second, the predictive performance of this model highly depends on the predicted secondary structures of pre-miRNA. Therefore, a feature encoding strategy that can combine several secondary structures given by quickfold or RNAFold for a pre-miRNA is desirable. Finally, the analyses of feature importance showed that the relational features that are localized in close proximity to the center of the pre-miRNA are more important than the other features. Accordingly, it might be potentially useful to consider more informative features close to the center of the pre-miRNA in future predictors.

## Supplementary Information

**Additional file 1.** Supplementary document.

## Data Availability

The dataset of this study are available at https://www.mirbase.org. The code implementing our method is available at https://github.com/ryuu90/ReCGBM.

## Abbreviations

Sn: sensitivity
Sp: specificity
Acc: accuracy
MCC: Matthews correlation coefficient
