# Supplementary material for: ReCGBM: a gradient boosting-based method for predicting human dicer cleavage sites
Source: BMC Bioinformatics. 2021 Feb 10;22:63. doi: 10.1186/s12859-021-03993-0 (PMC7877110; doi:10.1186/s12859-021-03993-0)
Supplement: Supplementary file 1 — Additional file 1. Supplementary document. [file 12859_2021_3993_MOESM1_ESM.pdf]

ReCGBM: a gradient boosting-based method for predicting human Dicer cleavage sites: supplementary document

Table S1: Performance comparison between different models based on the datasets with secondary structures predicted by quickfold. Best results are highlighted in bold. Sn, Sp, Acc and MCC represent sensitivity, specificity, accuracy and Matthews correlation coefficient, respectively

Method	5p-arm				3p-arm			
	Sn	Sp	Acc	MCC	Sn	Sp	Acc	MCC
ReCGBM	0.863	**0.846**	**0.854**	**0.709**	**0.883**	**0.899**	**0.891**	**0.783**
PHDCleav	0.853	0.831	0.842	0.685	0.875	0.874	0.874	0.749
LBSizeCleav (k=1)	0.865	0.823	0.844	0.689	0.869	0.877	0.873	0.747
LBSizeCleav (k=2)	0.864	0.828	0.846	0.693	0.869	0.874	0.871	0.744
LBSizeCleav (k=3)	**0.870**	0.822	0.846	0.693	0.863	0.871	0.867	0.735
LBSizeCleav (k=4)	0.863	0.822	0.842	0.686	0.851	0.868	0.860	0.720
LBSizeCleav (k=5)	0.863	0.819	0.841	0.684	0.854	0.868	0.861	0.723

Table S2: Performance comparison between different models based on the datasets with secondary structures predicted by RNAFold. Best results are highlighted in bold. Sn, Sp, Acc and MCC represent sensitivity, specificity, accuracy and Matthews correlation coefficient, respectively

Method	5p-arm				3p-arm			
	Sn	Sp	Acc	MCC	Sn	Sp	Acc	MCC
ReCGBM	0.884	**0.862**	**0.873**	**0.747**	0.888	**0.892**	0.890	0.781
PHDCleav	0.878	0.845	0.862	0.724	**0.904**	0.884	**0.894**	**0.789**
LBSizeCleav (k=1)	0.886	0.855	0.871	0.743	0.895	0.885	0.890	0.780
LBSizeCleav (k=2)	0.887	0.847	0.867	0.736	0.890	0.879	0.885	0.770
LBSizeCleav (k=3)	**0.888**	0.837	0.863	0.727	0.890	0.877	0.883	0.767
LBSizeCleav (k=4)	0.876	0.844	0.860	0.721	0.892	0.878	0.885	0.770
LBSizeCleav (k=5)	0.879	0.831	0.855	0.712	0.883	0.874	0.879	0.758

Table S3: Performance comparison between RECGBM with different secondary structures. Sn, Sp, Acc and MCC represent sensitivity, specificity, accuracy and Matthews correlation coefficient, respectively

Structures	5p-arm				3p-arm			
	Sn	Sp	Acc	MCC	Sn	Sp	Acc	MCC
quickfold	0.855	0.862	0.858	0.717	0.881	0.890	0.885	0.771
RNAFold	0.879	0.869	0.874	0.749	0.901	0.895	0.898	0.797
RNAstructure	0.874	0.871	0.872	0.745	0.896	0.897	0.897	0.794

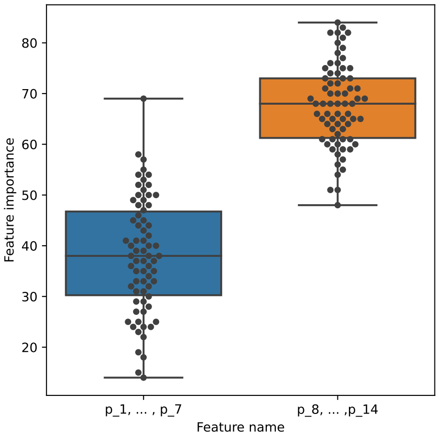

(a)

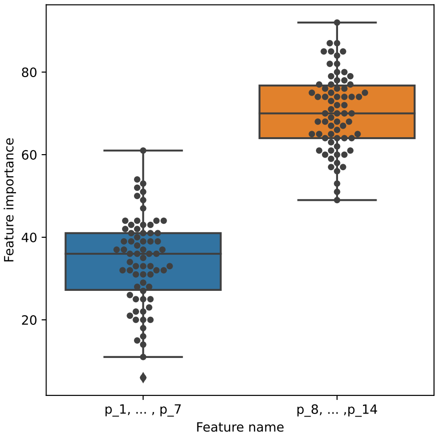

(b)

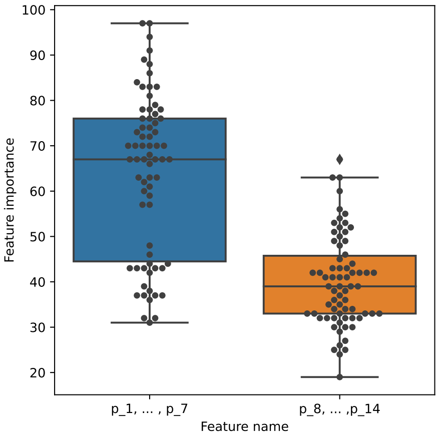

(c)

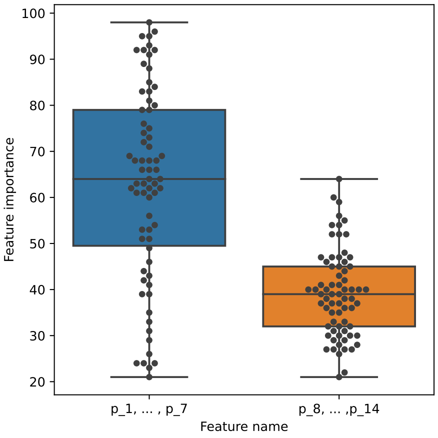

(d)

Figure S1: Comparisons of feature importance between $p_1, \ldots, p_7$ and $p_8, \ldots, p_14$. (a) is the result on 5p-arm data with secondary structures predicted by quickfold; (b) is the result on 5p-arm data with secondary structures predicted by RNAFold; (c) is the result on 3p-arm data with secondary structures predicted by quickfold; (d) is the result on 3p-arm data with secondary structures predicted by RNAFold.

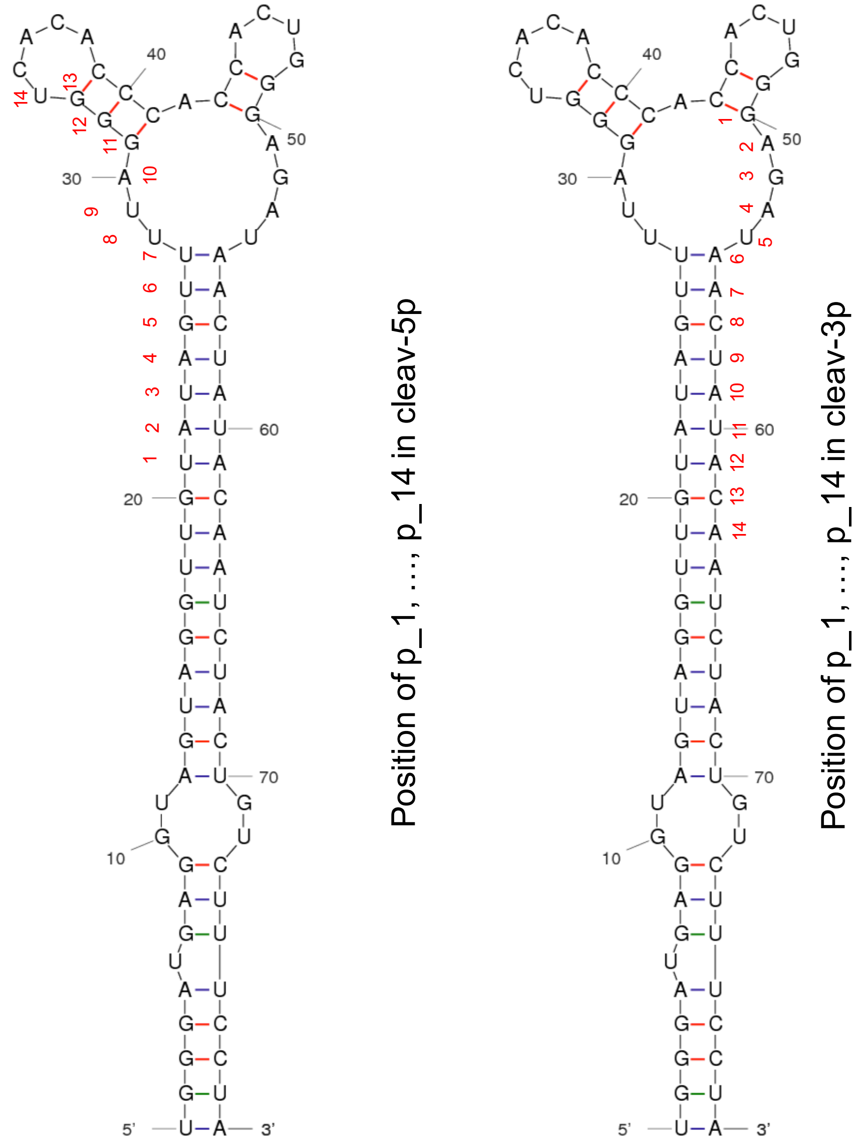

Figure S2: Positions of relational features in the secondary structure of pre-miRNA
